# Dimethyl­bis(3-methylsulfanyl-1,2,4-thia­diazole-5-thiol­ato)tin(IV)

**DOI:** 10.1107/S1600536809030062

**Published:** 2009-08-08

**Authors:** Junhong Zhang, Rufen Zhang, Chunlin Ma, Haizeng Wang, Daqi Wang

**Affiliations:** a College of Chemistry and Chemical Engineering, Ocean University of China, Qingdao 266100, Shandong, People’s Republic of China; b College of Chemistry and Chemical Engineering, Liaocheng University, Shandong 252059, People’s Republic of China

## Abstract

In the title compound, [Sn(CH_3_)_2_(C_3_H_3_N_2_S_3_)_2_], the Sn^IV^ atom is coordinated within a C_2_N_2_S_2_ donor set that defines a skew-trapezoidal bipyramidal geometry in which the methyl groups lie over the weakly coordinated N atoms. Two independent mol­ecules comprise the asymmetric unit, each of which lies on a mirror plane that passes through the C_2_Sn unit.

## Related literature

For related structures, see: Ma *et al.* (2005 ▶).
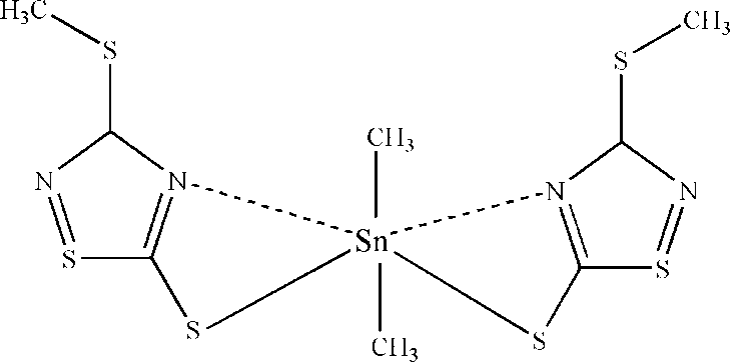


## Experimental

### 

#### Crystal data

[Sn(CH_3_)_2_(C_3_H_3_N_2_S_3_)_2_]
*M* *_r_* = 475.27
Orthorhombic, 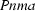

*a* = 13.721 (9) Å
*b* = 16.383 (10) Å
*c* = 16.282 (10) Å
*V* = 3660 (4) Å^3^
*Z* = 8
Mo *K*α radiation
μ = 2.07 mm^−1^
*T* = 293 K
0.48 × 0.37 × 0.25 mm

#### Data collection

Siemens SMART CCD area-detector diffractometer
Absorption correction: multi-scan (*SADABS*; Sheldrick, 1996 ▶) *T*_min_ = 0.436, *T*_max_ = 0.625
18306 measured reflections
3368 independent reflections
2269 reflections with *I* > 2σ(*I*)
*R*_int_ = 0.086

#### Refinement

*R*[*F* ^2^ > 2σ(*F* ^2^)] = 0.039
*wR*(*F* ^2^) = 0.107
*S* = 1.09
3368 reflections
181 parameters
H-atom parameters constrained
Δρ_max_ = 0.72 e Å^−3^
Δρ_min_ = −0.58 e Å^−3^

### 

Data collection: *SMART* (Siemens, 1996 ▶); cell refinement: *SAINT* (Siemens, 1996 ▶); data reduction: *SAINT*; program(s) used to solve structure: *SHELXS97* (Sheldrick, 2008 ▶); program(s) used to refine structure: *SHELXL97* (Sheldrick, 2008 ▶); molecular graphics: *SHELXTL* (Sheldrick, 2008 ▶); software used to prepare material for publication: *SHELXTL*.

## Supplementary Material

Crystal structure: contains datablocks I, global. DOI: 10.1107/S1600536809030062/tk2508sup1.cif

Structure factors: contains datablocks I. DOI: 10.1107/S1600536809030062/tk2508Isup2.hkl

Additional supplementary materials:  crystallographic information; 3D view; checkCIF report

## Figures and Tables

**Fig. 1.**
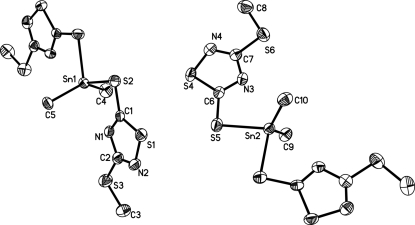
The molecular structure of (I), showing 30% probability displacement ellipsoids and atom labelling.

## supplementary crystallographic information

### Comment

Two independent molecules of (CH_3_)_2_Sn(C_3_H_3_S_3_N_2_)_2_ (I) comprise the asymmetric unit, each of which has mirror symmetry so that the C_2_Sn units lie on a plane (Fig. 1). The molecules have essentially equivalent bond distances and angles. Each tin atoms exists within a C_2_N_2_S_2_ donor set that defines a skew-trapezoidal bipyramidal geometry where the methyl groups lie over the weakly coordinated N atoms. The structure of (I) resembles closely those reported for a series of diorganotin(IV) 2-mercapto-4-methylpyrimidine derivatives (Ma *et al.*, 2005).

### Experimental

3-Methylmercapto-5-mercapto-1,2,4-thiadiazole (2 mmol) was added to a solution of ethanol (20 ml) containing sodium ethoxide (2 mmol). The mixture was stirred for 30 minutes after which dimethyltin dichloride (1 mmol) was added. Stirring continued  for 12 h at 318 K. After cooling to room temperature, the solution was filtered. The solvent was gradually removed by evaporation under vacuum until a solid product was obtained. The solid was then recrystallized from ether-dichloromethane to yield coulorless crystals; m. p. 356 K. Analysis, calculated for C_8_H_12_N_4_S_6_Sn: C 20.22, H 2.54, N 11.79; Found: C 20.16, H 2.49, N 11.83%.

### Refinement

All H atoms were placed geometrically and treated as riding on their parent atoms with C—H = 0.96 Å, and with  *U*_iso_(H) = 1.5*U*_eq_(C)].

### Crystal data

**Table:**

[Sn(CH_3_)_2_(C_3_H_3_N_2_S_3_)_2_]	*F*(000) = 1872
*M**_r_* = 475.27	*D*_x_ = 1.725 Mg m^−^^3^
Orthorhombic, *P**n**m**a*	Mo *K*α radiation, λ = 0.71073 Å
Hall symbol: -P 2ac 2n	Cell parameters from 5224 reflections
*a* = 13.721 (9) Å	θ = 3.0–24.2°
*b* = 16.383 (10) Å	µ = 2.07 mm^−^^1^
*c* = 16.282 (10) Å	*T* = 293 K
*V* = 3660 (4) Å^3^	Block, colourless
*Z* = 8	0.48 × 0.37 × 0.25 mm

### Data collection

**Table:**

Siemens SMART CCD area-detector diffractometer	3368 independent reflections
Radiation source: fine-focus sealed tube	2269 reflections with *I* > 2σ(*I*)
graphite	*R*_int_ = 0.086
φ and ω scans	θ_max_ = 25.0°, θ_min_ = 1.9°
Absorption correction: multi-scan (*SADABS*; Sheldrick, 1996)	*h* = −10→16
*T*_min_ = 0.436, *T*_max_ = 0.625	*k* = −19→19
18306 measured reflections	*l* = −17→19

### Refinement

**Table:**

Refinement on *F*^2^	Primary atom site location: structure-invariant direct methods
Least-squares matrix: full	Secondary atom site location: difference Fourier map
*R*[*F*^2^ > 2σ(*F*^2^)] = 0.039	Hydrogen site location: inferred from neighbouring sites
*wR*(*F*^2^) = 0.107	H-atom parameters constrained
*S* = 1.09	*w* = 1/[σ^2^(*F*_o_^2^) + (0.0351*P*)^2^ + 5.554*P*] where *P* = (*F*_o_^2^ + 2*F*_c_^2^)/3
3368 reflections	(Δ/σ)_max_ < 0.001
181 parameters	Δρ_max_ = 0.72 e Å^−^^3^
0 restraints	Δρ_min_ = −0.58 e Å^−^^3^

### Special details

**Table:**

Geometry. All e.s.d.'s (except the e.s.d. in the dihedral angle between two l.s. planes) are estimated using the full covariance matrix. The cell e.s.d.'s are taken into account individually in the estimation of e.s.d.'s in distances, angles and torsion angles; correlations between e.s.d.'s in cell parameters are only used when they are defined by crystal symmetry. An approximate (isotropic) treatment of cell e.s.d.'s is used for estimating e.s.d.'s involving l.s. planes.
Refinement. Refinement of *F*^2^ against ALL reflections. The weighted *R*-factor *wR* and goodness of fit *S* are based on *F*^2^, conventional *R*-factors *R* are based on *F*, with *F* set to zero for negative *F*^2^. The threshold expression of *F*^2^ > σ(*F*^2^) is used only for calculating *R*-factors(gt) *etc*. and is not relevant to the choice of reflections for refinement. *R*-factors based on *F*^2^ are statistically about twice as large as those based on *F*, and *R*- factors based on ALL data will be even larger.

### Fractional atomic coordinates and isotropic or equivalent isotropic displacement parameters (Å^2^)

**Table:**

	*x*	*y*	*z*	*U*_iso_*/*U*_eq_	Occ. (<1)
Sn1	0.82279 (4)	0.2500	0.19604 (3)	0.04949 (19)	
Sn2	0.43193 (4)	0.7500	0.09775 (3)	0.04987 (19)	
N1	0.8651 (4)	0.4226 (3)	0.1828 (3)	0.0512 (12)	
N2	0.8588 (4)	0.5637 (3)	0.1905 (3)	0.0652 (15)	
N3	0.3945 (4)	0.5731 (3)	0.0784 (3)	0.0523 (12)	
N4	0.4114 (4)	0.4321 (3)	0.0854 (3)	0.0636 (14)	
S1	0.75305 (13)	0.53359 (10)	0.23249 (11)	0.0635 (5)	
S2	0.70308 (12)	0.35277 (9)	0.24329 (11)	0.0622 (5)	
S3	1.02182 (15)	0.49793 (12)	0.12428 (12)	0.0799 (6)	
S4	0.51099 (13)	0.46584 (10)	0.13109 (10)	0.0637 (5)	
S5	0.54857 (14)	0.64762 (10)	0.14981 (11)	0.0685 (5)	
S6	0.25017 (14)	0.49009 (14)	0.00916 (12)	0.0833 (6)	
C1	0.7797 (4)	0.4321 (3)	0.2172 (3)	0.0487 (14)	
C2	0.9065 (5)	0.4983 (4)	0.1694 (4)	0.0568 (16)	
C3	1.0417 (6)	0.6055 (5)	0.1120 (5)	0.093 (3)	
H3A	1.1042	0.6144	0.0871	0.140*	
H3B	1.0398	0.6316	0.1647	0.140*	
H3C	0.9917	0.6279	0.0775	0.140*	
C4	0.8323 (8)	0.2500	0.0646 (5)	0.079 (3)	
H4A	0.7678	0.2500	0.0416	0.119*	
H4B	0.8666	0.2022	0.0467	0.119*	0.50
H4C	0.8666	0.2978	0.0467	0.119*	0.50
C5	0.9452 (7)	0.2500	0.2736 (6)	0.072 (3)	
H5A	0.9242	0.2500	0.3299	0.108*	
H5B	0.9837	0.2978	0.2632	0.108*	0.50
H5C	0.9837	0.2022	0.2632	0.108*	0.50
C6	0.4790 (4)	0.5664 (3)	0.1173 (3)	0.0486 (14)	
C7	0.3603 (5)	0.4953 (4)	0.0620 (3)	0.0525 (15)	
C8	0.2309 (6)	0.3820 (5)	0.0082 (5)	0.096 (3)	
H8A	0.1711	0.3700	−0.0198	0.144*	
H8B	0.2272	0.3623	0.0637	0.144*	
H8C	0.2841	0.3559	−0.0196	0.144*	
C9	0.4396 (8)	0.7500	−0.0320 (5)	0.066 (3)	
H9A	0.5066	0.7500	−0.0488	0.099*	
H9B	0.4078	0.7978	−0.0530	0.099*	0.50
H9C	0.4078	0.7022	−0.0530	0.099*	0.50
C10	0.3045 (7)	0.7500	0.1705 (6)	0.072 (3)	
H10A	0.3222	0.7500	0.2276	0.108*	
H10B	0.2667	0.7022	0.1586	0.108*	0.50
H10C	0.2667	0.7978	0.1586	0.108*	0.50

### Atomic displacement parameters (Å^2^)

**Table:**

	*U*^11^	*U*^22^	*U*^33^	*U*^12^	*U*^13^	*U*^23^
Sn1	0.0439 (3)	0.0543 (3)	0.0502 (3)	0.000	−0.0037 (3)	0.000
Sn2	0.0534 (4)	0.0459 (3)	0.0503 (3)	0.000	−0.0019 (3)	0.000
N1	0.051 (3)	0.048 (3)	0.054 (3)	0.008 (2)	−0.004 (3)	0.006 (2)
N2	0.080 (4)	0.048 (3)	0.067 (3)	−0.010 (3)	−0.015 (3)	0.009 (3)
N3	0.058 (3)	0.049 (3)	0.050 (3)	0.010 (2)	0.000 (3)	0.002 (2)
N4	0.077 (4)	0.054 (3)	0.060 (3)	−0.001 (3)	0.001 (3)	0.003 (2)
S1	0.0649 (11)	0.0480 (9)	0.0776 (11)	0.0094 (8)	−0.0071 (9)	−0.0073 (8)
S2	0.0522 (10)	0.0504 (9)	0.0841 (12)	0.0054 (8)	0.0027 (9)	0.0019 (8)
S3	0.0784 (13)	0.0800 (13)	0.0814 (13)	−0.0037 (11)	0.0119 (11)	0.0128 (10)
S4	0.0728 (11)	0.0481 (9)	0.0703 (10)	0.0163 (9)	−0.0074 (9)	0.0044 (8)
S5	0.0706 (12)	0.0535 (10)	0.0813 (12)	0.0090 (9)	−0.0135 (10)	0.0023 (8)
S6	0.0677 (12)	0.1003 (15)	0.0819 (13)	−0.0007 (11)	−0.0121 (11)	0.0127 (11)
C1	0.048 (4)	0.044 (3)	0.054 (3)	0.004 (3)	−0.014 (3)	0.003 (3)
C2	0.066 (4)	0.056 (4)	0.048 (3)	−0.001 (3)	−0.009 (3)	0.001 (3)
C3	0.086 (6)	0.091 (6)	0.102 (6)	−0.019 (5)	0.005 (5)	0.031 (5)
C4	0.086 (8)	0.110 (8)	0.043 (5)	0.000	0.011 (5)	0.000
C5	0.061 (6)	0.061 (6)	0.094 (7)	0.000	−0.032 (6)	0.000
C6	0.053 (4)	0.050 (3)	0.043 (3)	0.009 (3)	0.010 (3)	0.004 (3)
C7	0.059 (4)	0.066 (4)	0.033 (3)	−0.001 (3)	0.010 (3)	0.006 (3)
C8	0.092 (6)	0.108 (6)	0.088 (5)	−0.025 (5)	−0.009 (5)	0.006 (5)
C9	0.089 (7)	0.061 (6)	0.047 (5)	0.000	−0.004 (5)	0.000
C10	0.078 (7)	0.059 (6)	0.080 (6)	0.000	0.022 (6)	0.000

### Geometric parameters (Å, °)

**Table:**

Sn1—C5	2.102 (9)	S5—C6	1.721 (6)
Sn1—C4	2.144 (8)	S6—C7	1.741 (7)
Sn1—S2^i^	2.475 (2)	S6—C8	1.790 (8)
Sn1—S2	2.475 (2)	C3—H3A	0.9600
Sn1—N1	2.894 (5)	C3—H3B	0.9600
Sn2—C10	2.112 (9)	C3—H3C	0.9600
Sn2—C9	2.115 (8)	C4—H4A	0.9600
Sn2—S5^ii^	2.468 (2)	C4—H4B	0.9600
Sn2—S5	2.468 (2)	C4—H4C	0.9600
N1—C1	1.308 (7)	C5—H5A	0.9600
N1—C2	1.382 (7)	C5—H5B	0.9600
N2—C2	1.301 (8)	C5—H5C	0.9600
N2—S1	1.678 (6)	C8—H8A	0.9600
N3—C6	1.325 (7)	C8—H8B	0.9600
N3—C7	1.384 (7)	C8—H8C	0.9600
N4—C7	1.306 (7)	C9—H9A	0.9600
N4—S4	1.651 (6)	C9—H9B	0.9600
S1—C1	1.721 (6)	C9—H9C	0.9600
S2—C1	1.725 (6)	C10—H10A	0.9600
S3—C2	1.744 (7)	C10—H10B	0.9600
S3—C3	1.794 (8)	C10—H10C	0.9600
S4—C6	1.719 (6)		
			
C5—Sn1—C4	123.5 (4)	H3A—C3—H3C	109.5
C5—Sn1—S2^i^	110.1 (2)	H3B—C3—H3C	109.5
C4—Sn1—S2^i^	110.5 (2)	Sn1—C4—H4A	109.5
C5—Sn1—S2	110.1 (2)	Sn1—C4—H4B	109.5
C4—Sn1—S2	110.5 (2)	H4A—C4—H4B	109.5
S2^i^—Sn1—S2	85.74 (9)	Sn1—C4—H4C	109.5
C5—Sn1—N1	83.37 (11)	H4A—C4—H4C	109.5
C4—Sn1—N1	85.03 (11)	H4B—C4—H4C	109.5
S2^i^—Sn1—N1	145.17 (11)	Sn1—C5—H5A	109.5
S2—Sn1—N1	59.45 (11)	Sn1—C5—H5B	109.5
C10—Sn2—C9	127.0 (4)	H5A—C5—H5B	109.5
C10—Sn2—S5^ii^	110.1 (2)	Sn1—C5—H5C	109.5
C9—Sn2—S5^ii^	108.1 (2)	H5A—C5—H5C	109.5
C10—Sn2—S5	110.1 (2)	H5B—C5—H5C	109.5
C9—Sn2—S5	108.1 (2)	N3—C6—S4	111.4 (4)
S5^ii^—Sn2—S5	85.61 (10)	N3—C6—S5	124.6 (4)
C1—N1—C2	109.2 (5)	S4—C6—S5	124.0 (4)
C1—N1—Sn1	84.5 (3)	N4—C7—N3	119.4 (6)
C2—N1—Sn1	166.2 (4)	N4—C7—S6	124.8 (5)
C2—N2—S1	107.5 (4)	N3—C7—S6	115.8 (5)
C6—N3—C7	108.2 (5)	S6—C8—H8A	109.5
C7—N4—S4	108.1 (4)	S6—C8—H8B	109.5
N2—S1—C1	92.3 (3)	H8A—C8—H8B	109.5
C1—S2—Sn1	91.8 (2)	S6—C8—H8C	109.5
C2—S3—C3	100.4 (4)	H8A—C8—H8C	109.5
N4—S4—C6	92.9 (3)	H8B—C8—H8C	109.5
C6—S5—Sn2	93.5 (2)	Sn2—C9—H9A	109.5
C7—S6—C8	100.4 (4)	Sn2—C9—H9B	109.5
N1—C1—S1	111.6 (4)	H9A—C9—H9B	109.5
N1—C1—S2	124.2 (4)	Sn2—C9—H9C	109.5
S1—C1—S2	124.2 (4)	H9A—C9—H9C	109.5
N2—C2—N1	119.4 (6)	H9B—C9—H9C	109.5
N2—C2—S3	124.8 (5)	Sn2—C10—H10A	109.5
N1—C2—S3	115.9 (5)	Sn2—C10—H10B	109.5
S3—C3—H3A	109.5	H10A—C10—H10B	109.5
S3—C3—H3B	109.5	Sn2—C10—H10C	109.5
H3A—C3—H3B	109.5	H10A—C10—H10C	109.5
S3—C3—H3C	109.5	H10B—C10—H10C	109.5

Symmetry codes: (i) *x*, −*y*+1/2, *z*; (ii) *x*, −*y*+3/2, *z*.
